# A study of prevalence and distribution of tooth agenesis

**Published:** 2014

**Authors:** A Bozga, RP Stanciu, D Mănuc

**Affiliations:** * Clinic of Orthodontics and Dentofacial Orthopedics, “Carol Davila” University of Medicine and Pharmacy, Bucharest; ** Public Health Department, “Carol Davila” University of Medicine and Pharmacy, Bucharest

**Keywords:** dental agenesis, incidence, report, population

## Abstract

**Introduction.** Tooth agenesis is a phenomenon that occurs relatively commonly. The incidence of the missing teeth presented in the previous reports varies according to the studied population.

**Objective.** The aim of this study was to find the prevalence of tooth agenesis in a population group in Bucharest.

**Methods and results.** The prevalence and distribution of dental agenesis was determined in a sample of 518 patients, 285 females and 233 males, aged 6 to 41 years, who had been treated in the Clinic of Orthodontics and Dentofacial Orthopedics in Bucharest. The tooth agenesis was diagnosed by using the orthodontic records and study casts for each patient.

35 of the patients, 17 males and 18 females, were diagnosed with at least one absent permanent tooth and 47 missing permanent teeth were reported. A prevalence of 6.757% was observed for tooth agenesis. The mandibular second premolar was found to be the most affected tooth, followed by the maxillary lateral incisor, maxillary second premolar, mandibular central incisors, mandibular second molar and mandibular lateral incisor.

**Discussion.** The incidence of dental agenesis, its pattern and distribution per tooth type are in accordance with the previous published studies.

## Introduction

The previous studies reported for dental agenesis prevalence vary from 2.2 to 10.1%, most of them ranging between 6-8% [**1**-**8**,**11**,**18**,**22**]. The pattern and distribution of the congenitally absent teeth depend on the population investigated [**26**]: in Chinese and Japanese populations the mandibular central incisor is more commonly missing than in the Caucasian population [**9**,**12**,**16**].

The highest prevalence was reported in the Australian Caucasians 6.3%, followed by the European Caucasians 5.5% and the North American Caucasians 3.9% [**13**,**19**,**20**,**28**].

Other studies reported prevalence in different communities which vary from 2.6% in a population in South Arabia and 11.3% in an Irish population [**14**].

A meta-analysis made by Polder [**24**] in 2004, showed that the dental agenesis is usually 1.37 times more frequent in females than in males. The most affected teeth were found to be the mandibular second premolars, followed by the maxillary lateral incisors and the maxillary second premolars [**24**,**25**].

Firu [**10**] considers the small size or the absence of the maxillary lateral incisor as an evolutionary phenomenon.

The age when the tooth development usually takes place, but also the individual variations that can occur must be taken into account in diagnosing the congenital absence of teeth.

Although the beginning of the dental calcification is usually at 2-3 years old in premolars and permanent second molars (Logan and Krongfeldt, 1933, Schour and Massler, 1940, cited by Hölttä [**15**]), the mineralization of second premolars can take place even later [**21**,**23**]. This is the reason why, a correct diagnosis of tooth agenesis cannot be decided before the age of 6 in permanent dentition, if the third molars are not taken into consideration.

The etiology of tooth agenesis is usually genetic, the mode of inheritance being autosomal dominant in the majority of cases [**17**,**27**]. The differences reported in monozygotic twins also suggest environmental factors’ influence. Chemotherapy or radiotherapy, trauma, drugs or an infection (osteomyelitis, rubella) can affect the proliferation of the tooth bud cells.

Kjaer et al., cited by Wu [**28**] also associate mandibular tooth agenesis with nerve tissue, supporting tissues and oral mucosa disturbances.

## Methods

The study includes data obtained from a sample of 518 patients, 6 to 41 years of age, 285 females and 233 males, who referred to the Clinic of Orthodontics and Dentofacial Orthopedics in Bucharest, between the years 2007 and 2011. The orthodontic records of these patients: orthopantomogram, lateral cephalograms, diagnostic photographs and study casts, were analyzed. The age and sex of the patient and the number and distribution of the missing teeth were taken into consideration as well. The third molar was excluded from the present study.

## Results

**Fig. 1:**
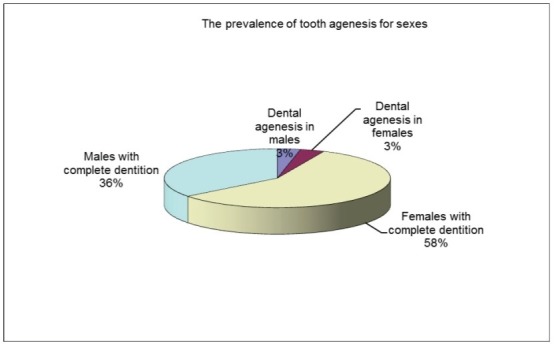
Tooth prevalence for both sexes

**Fig. 2:**
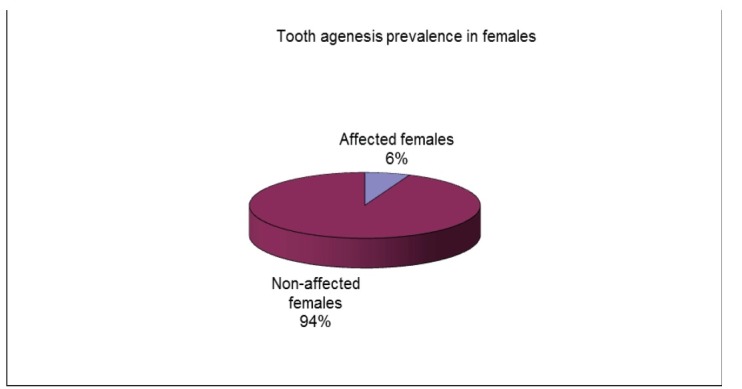
Tooth agenesis prevalence in females

**Fig. 3:**
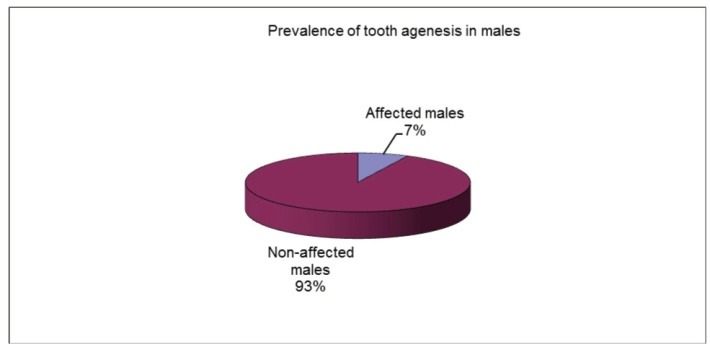
Tooth agenesis prevalence in males

Among the 518 patient records in this study, 35 subjects, 17 males (48.57%) and 18 females (51.43%) were diagnosed with permanent teeth agenesis. A prevalence of 6.757% was reported with dental disturbance. The prevalence found was of 6.31% for females and 7.29% for males (**Fig. 1**-**3**).

47 absent teeth were reported. The mandibular second premolar was found to be the most affected tooth (16 patients-19 missing teeth), followed by the maxillary lateral incisor (9 patients-11 absent teeth), maxillary second premolar (5 patients-7 missing teeth), mandibular central incisors (3 patients-6 absent teeth), mandibular second molar (2 patients-3 missing teeth) and mandibular lateral incisor (1 patient-1 absent tooth).

**Fig. 4:**
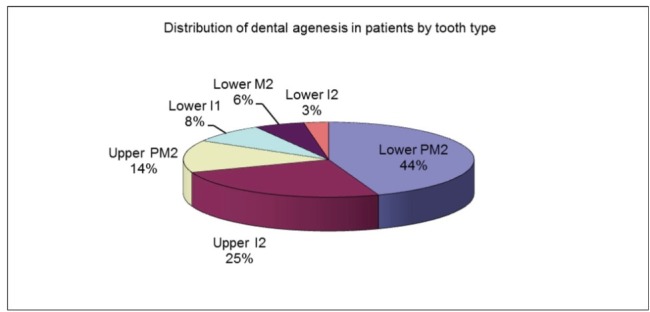
Distribution of dental agenesis in patients according to tooth type

**Fig. 5:**
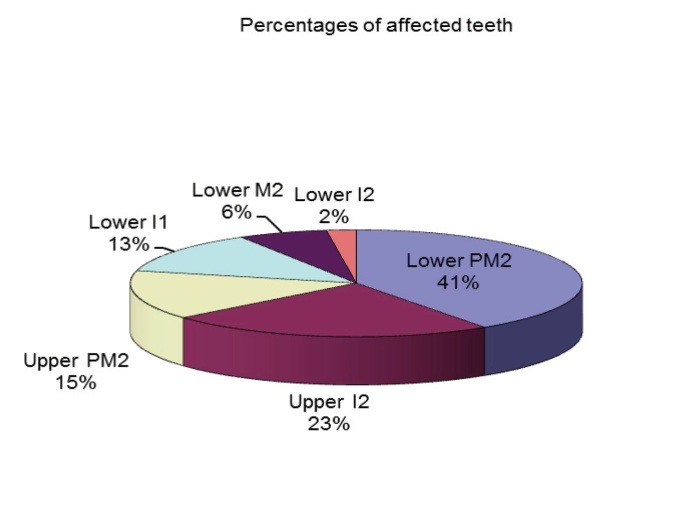
Percentages of affected teeth

The percentage of dental agenesis varied according to the tooth type (**Fig. 4**,**5**):

- 45.71% of the patients had at least one missing mandibular second premolar - 40.43% of the absent teeth were mandibular second premolars;

- 25.71% of the patients had at least one missing maxillary lateral incisor - 23.40% of the absent teeth were maxillary lateral incisors;

- 14.29% of the patients had at least one missing maxillary second premolar - 14.89% of the absent teeth were maxillary second premolars;

- 8.57% of the patients had at least one missing mandibular central incisor - 12.47% of the absent teeth were mandibular central incisors;

- 5.71% of the patients had at least one missing mandibular second molar - 6.38% of the absent teeth were mandibular second molars;

- 2.86% of the patients had at least one missing mandibular lateral incisor - 2.13% of the absent teeth were mandibular lateral incisors.

**Fig. 6:**
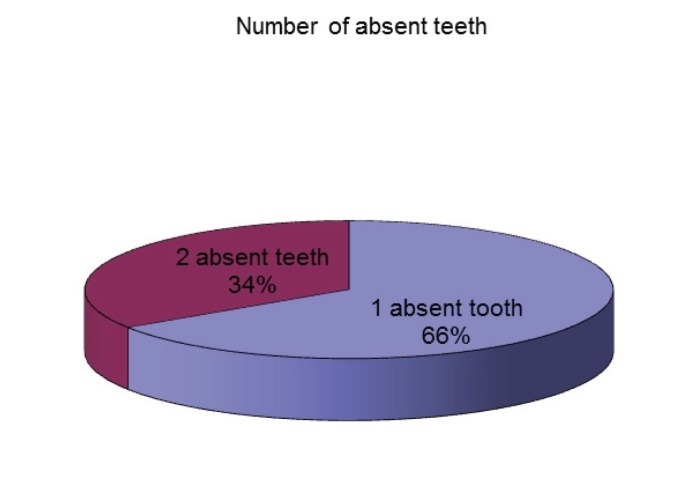
Number of absent teeth

**Fig. 7:**
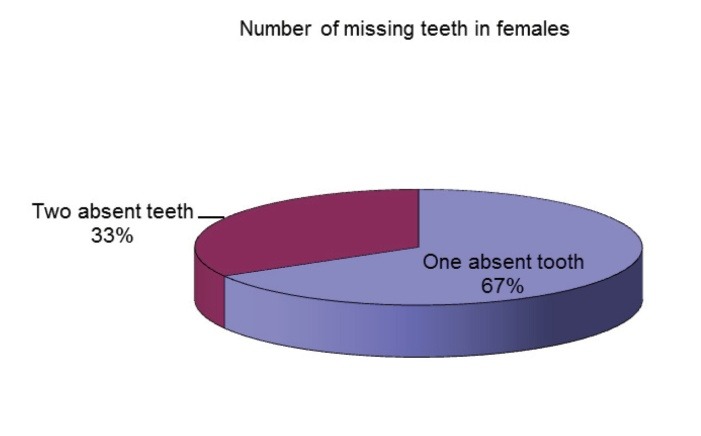
Number of missing teeth in females

**Fig. 8:**
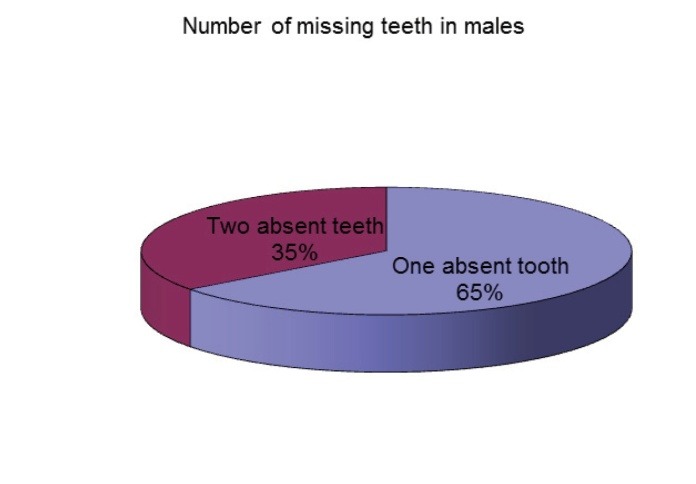
Number of missing teeth in males

Another notable issue is the fact that unilateral tooth agenesis is more common than the bilateral form and was found in 68.57% of the affected patients. 66% of the patients with tooth agenesis only had one absent tooth and 34% of them had two missing teeth (**Fig. 6**). There were no patients with the absence of more than two teeth in the studied sample. The absence of two teeth was more frequent in males than in females (**Fig. 7**,**8**).

## Conclusions

The results of this study showed a prevalence of dental agenesis of 6.76%, which is in the range of values obtained from the previous reports.

The mandibular second premolar was the most affected tooth (45.71% of the patients and 40.43% of the absent teeth), followed by the maxillary lateral incisor, maxillary second premolar, mandibular central incisors, mandibular second molar and mandibular lateral incisor.

The pattern and distribution per tooth type were in accordance with the other published studies.
